# 3D cotton-type anisotropic biomimetic scaffold with low fiber motion electrospun via a sharply inclined array collector for induced osteogenesis

**DOI:** 10.1038/s41598-024-58135-2

**Published:** 2024-03-28

**Authors:** Sun Hee Cho, Soonchul Lee, Jeong In Kim

**Affiliations:** 1 Department of Bionanotechnology and Bioconvergence Engineering, Graduate School, Jeonbuk National University, Jeonju, 54896 Republic of Korea; 2 Department of Orthopaedic Surgery, CHA Bundang Medical Center, CHA University School of Medicine, 335 Pangyo-ro, Bundang-gu, Gyeonggi-do Republic of Korea

**Keywords:** Electrospinning, 3D fibers, Cottony fibers, Biomimetic scaffolds, Osteogenesis, Bone tissue engineering, Cell biology, Engineering, Materials science, Nanoscience and technology

## Abstract

Electrospinning is an effective method to fabricate fibrous scaffolds that mimic the ECM of bone tissue on a nano- to macro-scale. However, a limitation of electrospun fibrous scaffolds for bone tissue engineering is the structure formed by densely compacted fibers, which significantly impedes cell infiltration and tissue ingrowth. To address this problem, several researchers have developed numerous techniques for fabricating 3D fibrous scaffolds with customized topography and pore size. Despite the success in developing various 3D electrospun scaffolds based on fiber repulsion, the lack of contact points between fibers in those scaffolds has been shown to hinder cell attachment, migration, proliferation, and differentiation due to excessive movement of the fibers. In this article, we introduce a Dianthus caryophyllus-inspired scaffold fabricated using SIAC-PE, a modified collector under specific viscosity conditions of PCL/LA solution. The developed scaffold mimicking the structural similarities of the nature-inspired design presented enhanced cell proliferation, infiltration, and increased expression of bone-related factors by reducing fiber movements, presenting high space interconnection, high porosity, and controlled fiber topography.

## Introduction

A major goal of bone tissue engineering is to provide scaffolds that act as an ideal bone graft material for repairing bone defects caused by trauma, osteoporosis, infection, or tumors. A scaffold should be designed to mimic the extracellular matrix (ECM) of natural bone tissue with hierarchical nanocomposites and provide a microenvironment that possesses the appropriate topography and three-dimensional (3D) structure for efficient cell adhesion, proliferation, and osteogenic differentiation^1,2^. Therefore, the development of artificial bone scaffolds with well-defined pore structures and surface topographies that support cell functions is essential^3,4^. Kim et al.^5^ demonstrated a method to manipulate micro- and nano-patterned substrates using capillary force lithography and wrinkling techniques and found that hierarchical multiscale PLGA patches showed great potential for bone regeneration. However, the interactions between cells and scaffolds in many studies can still only be controlled at the substrate surface^6^. Cells demand a 3D space with interconnected pores to allow for migration into the interior of the scaffold following topographical cues. Zhang et al.^7^ utilized a ceramic 3D printing technology to prepare 3D macro/micro hierarchical scaffolds. 3D-printed bioceramic porous scaffolds allow cell penetration, adherence, growth, and proliferation that lead to bone growth. However, due to the limitations of printing technology and the hardness and brittleness of materials, it is still difficult to fabricate customized micro/nano topography. Numerous attempts have been made to recreate a fibrous structure mimicking the fibrous collagen network of native ECM bone^8,9^.

The fabrication of polymer fibers-based bone scaffolds is an attractive approach for the development of successful tissue-engineered scaffolds. Although the fabrication of polymeric fibers can be accomplished using methods such as phase separation and self-assembly, electrospinning has emerged as a versatile and cost-effective technique for the fabrication of nanofibers and microfibers that mimic the morphological properties of ECM^10,11^. Fibrous scaffolds fabricated by general electrospinning technology behave similarly to two-dimensional (2D) scaffolds with diameters in the range of 50–1000 nm, which have favorable structural and mechanical properties^12^. However, the limited thickness and pore size of the scaffolds can be disadvantages of conventional electrospinning. The shape of natural bone defects is complex and variable, and simple 2D electrospun mats may not meet the necessary requirements of a scaffold for bone regeneration.

**Figure 1:**
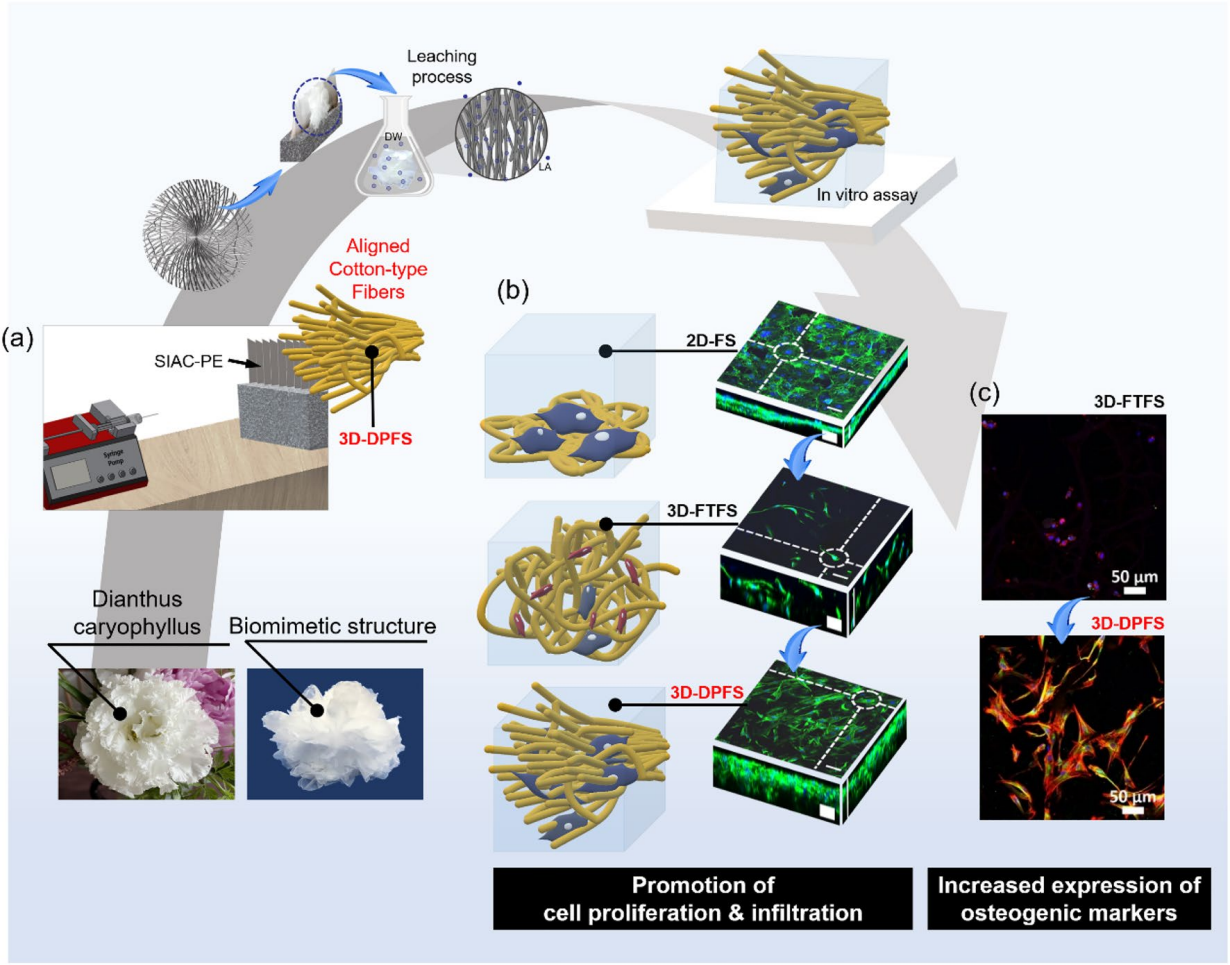
Schematic illustration showing (**a**) the fabrication method of 3D-DPFS with low fiber motion electrospun via a SIAC-PE for (**b**) promoted proliferation, infiltration, and (**c**) osteogenic differentiation of MC3T3-E1 cells.

In fact, the unique pore features of 3D structures such as high bulk porosity (up to 95%), isotropic structure, and homogeneous fiber size (1–1000 μm) eliminate many constraints of 2D structure for tissue growth and nutrient distribution^13^. Fortunately, in recent years, new technologies based on electrospinning have been applied to the fabrication of 3D nanofiber scaffolds^14,15^. Electrospun 3D nanofibrous matrices with high spatial interconnectivity, high fiber density, and controlled alignment have been well-studied to direct cell orientation and migration^16^. However, recent studies have attempted to manufacture 3D electrospun scaffolds based on fiber repulsion and have shown that excessive movement of the fibers caused by a lack of contact points between fibers hinders cell adhesion and elongation on the fibers^17,18^. Actually, in our findings, the activity of the cells is significantly lower from the cotton-type 3D fibrous scaffold than the 2D fibrous scaffold. To overcome the disadvantages of 3D fibrous scaffolds, we continued our efforts to obtain 3D fibrous scaffolds that reduced movement between fibers using the previously developed sharp inclined array collector with point electrode (SIAC-PE). As a result, we successfully generated a scaffold inspired by the Dianthus caryophyllus for bone tissue regeneration by utilizing SIAC-PE in a certain viscosity condition of the lactic acid (LA) blended polycaprolactone (PCL) solution. The developed scaffold mimicked the structural or aesthetic similarities of the nature-inspired design. This dianthus caryophyllus-mimicking 3D fibrous scaffold showed promoted cell proliferation, infiltration, and increased expression of bone-related factors by reducing fiber movements, presenting high space interconnection, high porosity, and controlled alignment (Fig. 1).

## Materials and methods

### Scaffold fabrication

A 10% (w/w) polycaprolactone (PCL, M_w_ = 80,000, Sigma-Aldrich Korea) solution was prepared by dissolving it in dichloromethane (DCM, Samchun, Korea) and *N*, *N*-dimethylformamide (DMF, Samchun, Korea) in a 4:1 ratio. Lactic acid (LA, Samchun, Korea) was then added to the polymer solution to a concentration of 18% (w/w). After 6 h of magnetic stirring, the solution was kept in room temperature for 3–4 days. The set up and parameters for electrospinning followed a process that had been described previously^19^. 2D membranes and 3D structures were deposited on the sharply inclined array collector with point electrode (SIAC-PE) by varying the temperature and relative humidity. 2D membranes were generated in a low temperature (≤ 19 °C) and relative humidity environment (≤ 20%), whereas 3D structures with aligned cotton-type fibers were fabricated in a relatively high temperature (≥ 23 °C) and relative humidity environment (≥ 40%). For the fabrication of conventional 3D electrospun scaffolds with high fiber motion as a control group, the same polymer solution was electrospun on a pin-type collector reported by Hwang et al.^20^. Electrospinning for the 3D control group was performed under the same conditions (40%, 25 °C) as for the 3D comparison group. All scaffolds fabricated under different condition were vigorously rinsed with distilled water (DW) to leach LA after electrospinning.

### Scaffold characterization

Viscosity of PCL/LA solution was measured by digital viscometer (Brookfield, DV-3+, USA). The morphology of the various samples was visualized by scanning electron microscopy (SEM, Hitachi, Japan). Image J (NIH, USA) was utilized to analyze the diameter, orientation distribution, and porosity from three FE-SEM images. The fiber alignment was determined by Fast Fourier Transform (FFT) analysis. The compressive strength of 3D-type samples was measured with a universal testing machine (UTM; MTDI INC., Korea) equipped with a 100 N load cell. The tensile tests for the three scaffolds were also performed by the UTM with a 10 N load sensor with a strain rate of 5 mm/min at room temperature.

### In vitro degradation study

The degradation profile of two 3D fibrous scaffolds was assessed by changes in the surface morphology of the scaffold. The initial samples were fully soaked in phosphate-buffered saline (PBS), and then incubated at 37 °C. The solution was refreshed every 2 days. Samples were taken out at different time points (7, 14, 21, and 28 days) for scaffolds soaked in PBS. The degradation rates were determined using the FE-SEM images.

### In vitro biomimetic mineralization study

The assessment of in vitro bio-mineralization of the three samples was carried out in simulated body fluid (SBF) solution with the following ion concentrations: 142 mM of Na^+^, 5.0 mM of K^+^, 1.5 mM of Mg^2+^, 2.5 mM of Ca^2+^, 147.8 mM of Cl^-^, 4.2 mM of HCO_3_^-^, 4.2 mM of HPO_4_^2-^, 0.5 mM of SO_4_^2-^ that included 50.0 mM trishydroxymethylaminomethane (Tris) and 45.0 mM hydrochloric acid (HCl). The SBF solution was prepared by dissolving the calculated amounts of NaCl, NaHCO_3_, KCl, K_2_HPO_4_∙3H_2_O, MgCl_2_∙6H_2_O, CaCl_2_, and Na_2_SO_4_ in distilled water with a stirring bar into 1000 ml glass beaker. The solution was buffered with Tris/HCl to pH 7.4 at 36.5 ℃. The samples were incubated with the SBF solution at 37 °C. The SBF solution was replaced every 48 h. SBF-treated samples were rinsed with distilled water and then dried at room temperature. An apatite-like crystals formation on the surface of the fibers was observed by FE-SEM imaging combined with EDS analysis. Alizarin red S (ARS) was further used to evaluate the decomposition of calcium compounds on the SBF treated samples; briefly, SBF-treated samples were washed with distilled water, then fixed in 3.7% buffered formaldehyde for 30 min, and stained with 1 ml of ARS solution (0.04 M, pH 4.1) in a 48-well plate for 20 min on a shaker. Samples were then rinsed with distilled water to remove the excess dye, transferred into another well plate, and treated with 50% acetic acid (1 ml) for 30 min. The dissolved dye was diluted with distilled water in a 1:4 ratio, and pH was adjusted to 4.1. A microplate reader (Sunrise Tecan, Austria) was used to measure the absorbance of the solution at 492 nm in a 96-well plate.

### Cell culture, proliferation, and morphology staining

Pre-osteoblast (MC3T3-E1) cells were purchased from ATCC (CRL-5293) to study the biocompatibility and cellular behavior of the scaffolds. Before cell seeding, the scaffolds were placed in a 48-well plate, sterilized under ultra-violet (UV) light for 24 h, and washed with ethanol and phosphate-buffered saline (PBS). Cells were seeded at a density of 3 × 10^4^ per well. The seeded cells were cultured in alpha modification of Minimum Essential Medium (α-MEM, Hyclone) containing 10% fetal bovine serum (FBS, Gibco) along with 1% penicillin–streptomycin (Gibco) in an incubator at 37 °C in a humidified atmosphere with 5% carbon dioxide (CO_2_). Fresh culture medium was given every other day.

The quantitative evaluation associated with the viability of MC3T3-E1 cells on different fibrous scaffolds was carried out using cell counting kit-8 (CCK-8, Dojindo Molecular Technologies, Inc. USA) after (1, 3, and 7) days. At set times, the medium was removed, and each well was replaced with a fresh medium with 10% CCK-8 reagent. After incubation for 2 h in a humidified incubator at 37 °C in the dark, a 200 μl aliquot of color-changed solution was transferred into a 96-well plate. The optical density (OD) was measured at 450 nm (n = 3) to examine the proliferation of cells using a spectrophotometric microplate reader (Tecan, Austria).

To observe the cell morphology, the cell-cultured scaffolds were carefully washed twice with PBS to remove unattached cells and fixed using 4% paraformaldehyde for 15 min. After complete removal of paraformaldehyde with PBS, the cells were permeabilized with 0.3% Triton X-100 for 3 min and blocked using 1% human serum albumin (HSA) for 30 min. Subsequently, the cells were stained with Actin-Green 488 (R37110, Thermo Fisher) and 4′,6-diamidino-2-phenylindole (DAPI, Thermo Fisher Scientific, USA) to cytoskeleton for 20 min and nuclei for 5 min, respectively, in dark condition. Finally, the samples were washed with PBS to remove excess stains, and fluorescence imaging was performed via confocal laser scanning microscopy (CLSM, LSM 800 Airyscan, Carl Zeiss, Germany). Further, the fluorescent images of the cultured cells throughout the scaffolds were analyzed via Z-stack orthogonal projections using ZEN Blue software.

### Immunofluorescence staining

To investigate the secretion of osteogenesis-related gene expression, MC3T3-E1 cells at a density of 3 × 10^4^ per well were cultured with various groups of samples for 3 days. The cultured MC3T3-E1 cells were fixed in 4% paraformaldehyde for 10 min, and washed twice with PBS at RT. The cells were then permeabilized in 0.3% Triton X-100 for 5 min, blocked with 1% HSA for 30 min, with subsequent incubation of the primary antibody at 4 °C overnight, and then labeled with secondary antibody for 1 h at RT in dark condition. The nuclei were counterstained with DAPI for 10 min. The samples were rinsed with PBS at each step. The stained cells were then examined using Super Resolution CLSM.

## Results and discussion

### Mechanism of fabrication of electrospun scaffolds mimicking dianthus caryophyllus

**Figure 2:**
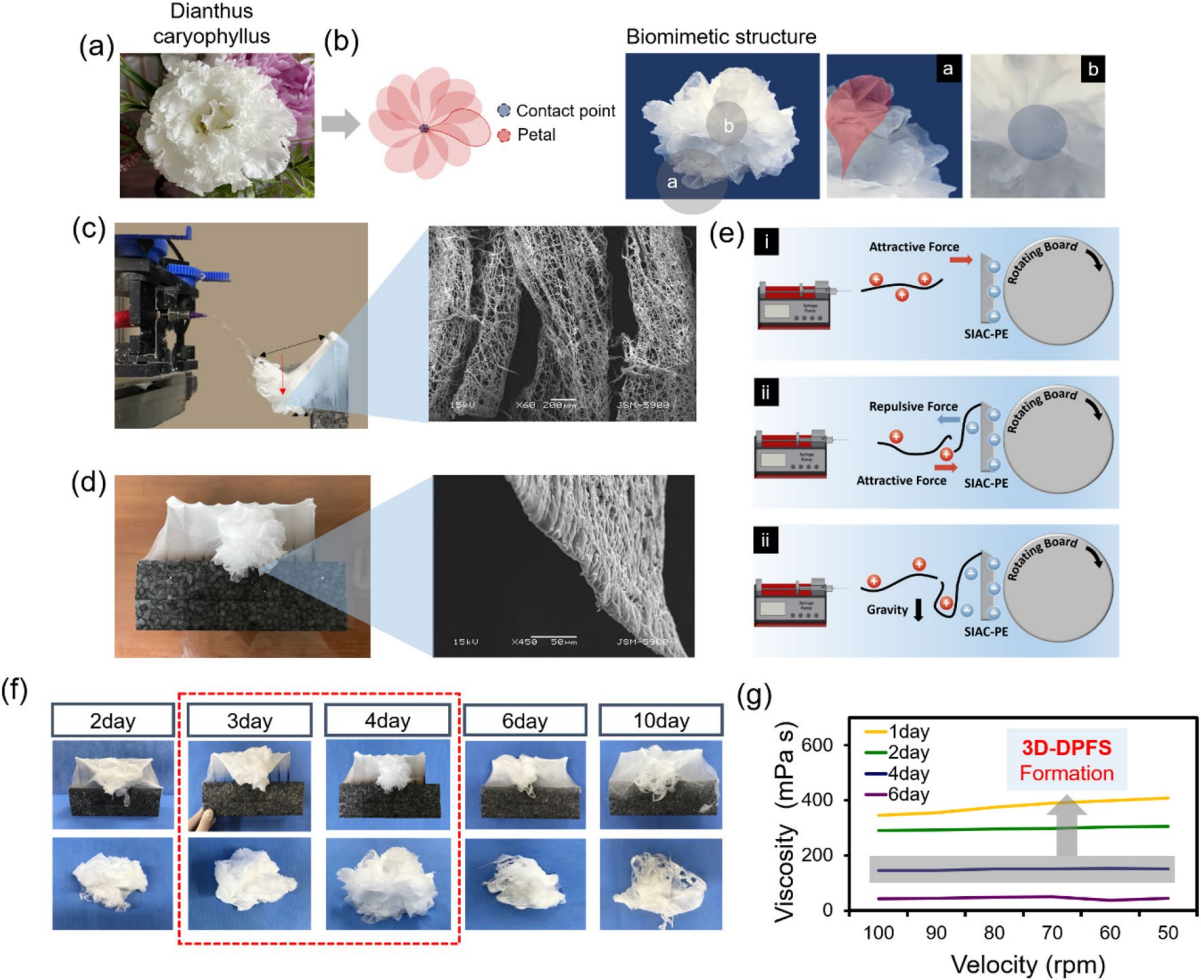
Forming structure and mechanism of 3D-DPFS. (**a**) Photograph of dianthus caryophyllus. (**b**) Illustration and photograph of 3D-DPFS with a nature-inspired angiosperm-like structure. (**c**) Photograph and SEM image showing the formation of hierarchically aligned cotton-type fibrous scaffolds during electrospinning. (**d**) Photograph and SEM image of fibrous scaffold. (**e**) Schematic illustrating how the electric field directs fiber stacking between the SIAC-PE. (**f**) Photographs of electrospun fibrous scaffolds with various shapes deposited on the SIAC-PE according to solution stirring times. (**g**) The viscosity of PCL/LA solution by increasing stirring time.

In a previous study, a sharply inclined array collector (SIAC) composed of nine edged bars and pedestal was designed. Setting the SIAC with a point electrode (SIAC-PE) led to the deposition of aligned cotton-type fibers (Fig. 1a). Verification of the mechanism of 3D electrospinning using LA assisted solution and SIAC-PE was discussed in detail in our previous research^19^. Figure 2a and b show photographs of the mimetic structure of Dianthus caryophyllus consisting of drooping, stacked electrospun fibers deposited directly on the SIAC-PE. SEM images taken from the same sample are also shown in Fig. 2c, d, confirming that the fibers simulated the macrostructure of the petal. Figure 2e briefly describes the principle of 3D scaffold generation of PCL/LA fibers during electrospinning. Among the point electrodes (PE) arranged in a row, the electric field concentrated on the central PE closest to the needle tip induces the deposition of the most fibers on the central PE. Within the strong electric field, the deposited fibers become negatively charged due to electrostatic force and polarization; thus, these fibers act as a new electrode to attract subsequently deposited fibers (Fig. 2e)^21^. Since the initial fibers gain the same charge as the electrode, they tend to repel against the electrode and form a loosely assembled structure. From there, the continuous overlapping of drooping fibers due to gravity contributed to the maintaining of the 3D shape as shown in Fig. 2c and d. Besides the aforementioned effect of the PE, the properties of the electrospinning solution had a significant impact on the sophisticated structural mimicry of Dianthus caryophyllus on the fibrous scaffold. The morphological alteration of electrospun fibers deposited on the SIAC-PE changed with increasing stirring time of a PCL/LA solution prior to electrospinning, as shown in Fig. 2f. The electrospun scaffolds perfectly simulated the shape of a dianthus caryophyllus when the PCL/LA solution was stirred for more than 72 h and less than 96 h (Fig. 2g). We found that the effective viscosity of the PCL/LA solution is crucial in designing 3D biomimetic structure in the modified electrospinning set up with SIAC-PE.

### Morphological properties of scaffolds

**Figure 3:**
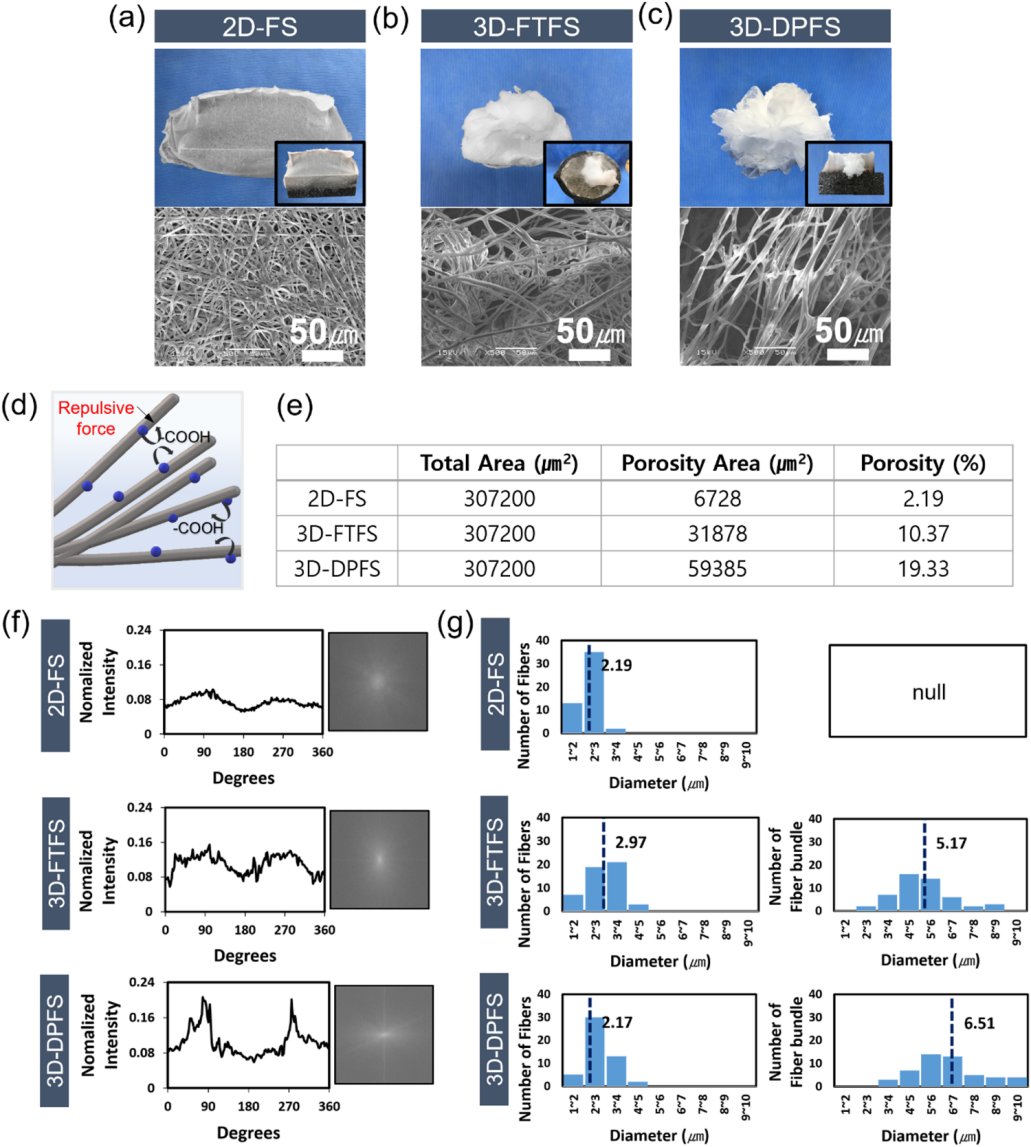
(**a**–**c**) Digital photos and SEM images of 2D-FS, 3D-FTFS, and 3D-DPFS deposited on customized collectors. (**d**) Illustration of LA plays in the electrospinning solution. (**e**) Porosity measurement results of SEM images (**a**–**c**). (**f**) FFT graphs and output images, (**g**) graphs of fiber and bundle type diameters of 2D-FS, 3D-FTFS, and 3D-DPFS.

Figure 3a–c depicts a thin sheet and two 3D structures of PCL fibers containing LA deposited on two different collectors. First, as shown in Supplementary Fig. 1a, b, a 2D PCL/LA fibrous scaffold (2D-FS) and a 3D dianthus caryophyllus-mimetic PCL/LA patterned fibrous scaffold (3D-DPFS) were fabricated via the SIAC-PE as previously reported^19^. Next, a fluffy-type 3D PCL/LA fibrous scaffold (3D-FTFS) was made on a spinner collector invented by Hwang et al.^20^ (Supplementary Fig. 1). Again, all solutions used for electrospinning were the same. SEM images of 2D-FS reveal tightly packed fibrous layers with low porosity (Fig. 3e), a typical phenomenon for randomly oriented electrospun membranes (Fig. 3a). It has been reported that this dense structure is not favorable for cell filtration, especially for scaffolds made from hydrophobic materials ^15^. On the other hand, 3D-FTFS and 3D-DPFS scaffolds consisted of non-dense structures with porosities of 10% and 19% (Fig. 3e), respectively. This result is presumably due to electrostatic repulsion between the carboxylic acid groups (–COOH) of LA in the adjacent fibers as presented in Fig. 3d^22,23^. Also, 3D-DPFS exhibited an aligned fiber topography, in contrast to 3D-FTFS, which consisted of randomly oriented fibers (Fig. 3b, c).

The structural properties of the three samples were further assessed via fiber diameter measurement and FFT analysis using the Image J program (Fig. 3f and g). The fiber orientations for the 2D-FS and 3D-DPFS, produced by SIAC-PE, were consistent with our previous study using the same electrospinning parameters, humidity, and room temperature. Likewise, 3D-FTFS prepared using the spinner collector exhibited random fiber orientation as reported in Hwang's previous findings (Fig. 3f). All electrospinning conditions, including solution stirring time, for 3D-FTFS and 3D-DPFS were perfectly matched. The average fiber diameters of 2D-FS and 3D-DPFS were 2.19 μm and 2.17 μm, while the average fiber diameter of 3D-FTFS was slightly higher at 2.97 μm (Fig. 3g). The 3D-DPFS and 3D-FTFS have a large number of fiber bundles, and the increased diameter due to the fiber bundling effect could be a factor in producing structures with high porosity^24^. The production of a three-dimensional porous fibrous network is an effective way to allow for cell infiltration^25^.

### Mechanical and degradation properties of scaffolds

**Figure 4:**
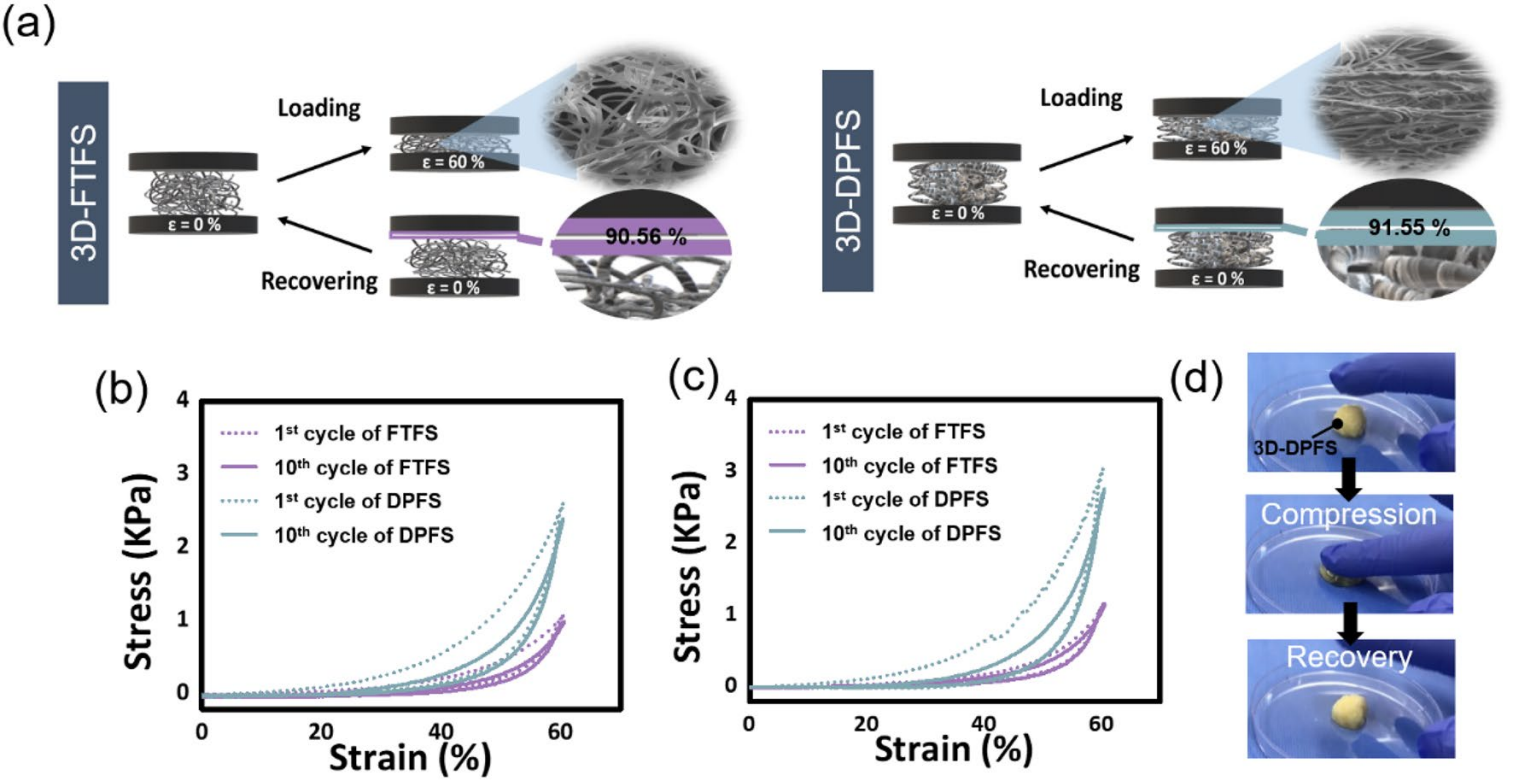
Comparison of mechanical properties of 3D-FTFS and 3D-DPFS. (**a**) Schematic illustration of 3D samples under loading and recovering cycles. Compressive stress–strain curves of 3D samples in (**b**) dry and (**c**) wet conditions. (**d**) Optical images of 3D-DPFS before and after compression in wet condition.

Bone is a dynamic tissue and applied loads play vital roles in determining the rate of turnover, the formation of callus, its volume, and stiffness during bone healing. It was reported that scaffolds possessing some elasticity provide a load-transducing environment in which matrix deposition, new bone formation, and maturation can take place^26,27^. The 3D-FTFS and 3D-DPFS exhibited elastic properties in dry and wet environments, respectively. Compressive stress–strain curves of 3D-FTFS and 3D-DPFS are shown in Fig. 4a–c, both exhibiting highly nonlinear and closed hysteresis. These two scaffolds could withstand 60% compressive strain and recovered their initial shape after compression (Fig. 4b and c). However, the maximum stress of 3D-FTFS was approximately two times lower than that of 3D-DPFS, in both dry and wet environments. Although there have been reports of a smaller difference in the mechanical property of PCL samples with increased porosity in a different study, the differences observed here may be attributed to topographical features such as pore structure and morphology (alignment) of the fibrous matrix^28^. As a result of evaluating the structure of the scaffolds before and after compression in a wet state, no significant contraction was observed in 3D-DPFS compared to other scaffolds even when an external force was applied (Fig. 4d). In addition, the maximum stresses of wet both samples increased from the 1st until the 10th cycle of the compression tests compared to the dry scaffolds. A plausible explanation for this behavior is the internal fibers of the scaffold sticking together after absorbing water; the higher the fiber density, the greater the number of fibers that stick together, hence the stronger the mechanical properties of the scaffold^29^. Finally, we conducted tensile tests to compare the mechanical properties of the thin sheet and two 3D structures as shown in Fig. S1. The tensile strength was 3.609 MPa, which higher than that of 3D-FTFS and 3D-DPFS (Fig. S1). The lower values of tensile strength of the two 3D structures are attributed to its highly stacked and porous nature^30^. Chen et al.^25^ reported that although the mechanical property of 3D cotton material was insufficient, it was greatly improved after subcutaneous implantation. Therefore, it can be asserted that this novel strategy for fabricating 3D scaffolds is a promising technique for preparing load-bearing ECM-like tissue engineering scaffolds.

The morphological structure of the as-spun fibrous scaffolds affects the mechanical properties and rate of degradation, enabling it to maintain the structural integrity and stability necessary for use in bone tissue engineering. The degradation behavior of the two 3D fibrous scaffolds was assessed using SEM images. The morphology of the scaffolds after immersion in PBS was evaluated every 7 days to check the degree of degradation for each scaffold type, as presented in Fig. S2. After degradation for 14 days, the fibers of 3D-FTFS started to fuse with each other, resulting in a state where the boundaries of the fibers could not be distinguished. On the other hand, the 3D-DPFS did not show any significant changes in fiber orientation or the overall structure after 7, 14, 21, and 28 days of degradation (Fig. S2).

### In vitro biomineralization properties of scaffolds

**Figure 5:**
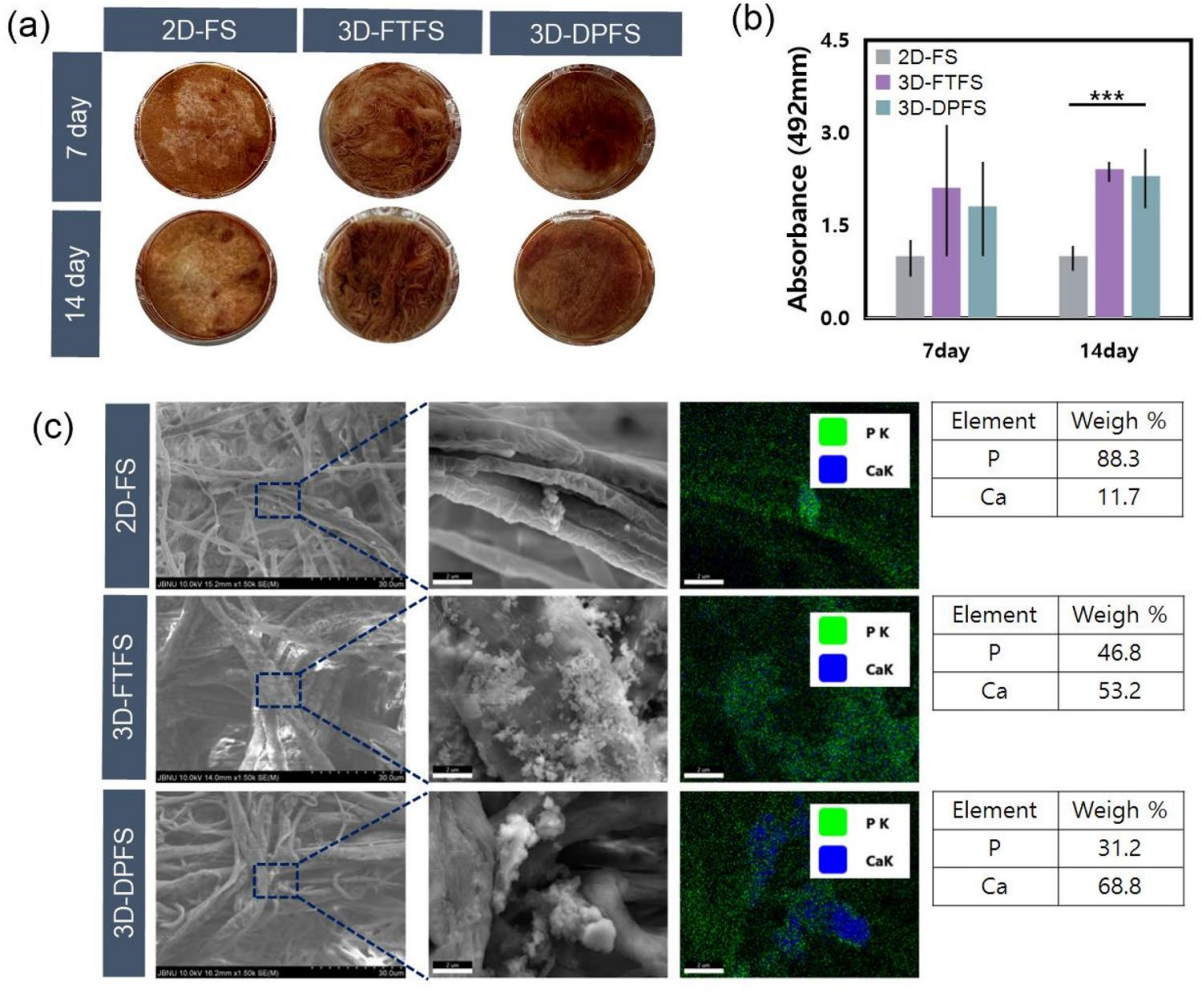
(**a**) The optical images of the mineralized scaffolds stained with ARS. (**b**) Quantitative analysis of calcium deposition on scaffolds through ARS staining. (**c**) FE-SEM images of HA deposited on scaffolds after soaking in SBF for 7 days. Right: comparison of Ca and P distribution results via EDS mapping in high-magnification images.

To improve the bone repair ability of the scaffolds, the in vitro biomineralization properties were evaluated by soaking the scaffolds in SBF ^1^. As shown in Fig. 5a–c, HA crystals formed in each sample starting after 7 days. In 2D-FS, some HA crystals were attached to the fiber surface, whereas in the two 3D structures, many HA crystals were generated in aggregate form along the fiber surface and pores (Fig. 5c). After soaking the scaffolds in SBF for 7 and 14 days, the deposition of calcium compounds was quantified based on Alizarin Red S (ARS) analysis as shown in Fig. 5b. The values of 3D-FTFS and 3D-DPFS were significantly higher than those of 2D-FS, indicating that the deposition of many calcium compounds can be accommodated on the 3D scaffold (Fig. 5b). Additionally, the amount of calcium compound deposition in 3D-FTFS and 3D-DPFS was similar. However, the essential elements (Ca, P) of HA were observed using EDS, and the Ca/P ratio for 3D-DPFS was most similar to hydroxyapatite (Ca/P ratio near 1.67) compared to 3D-FTFS (Fig. 5c)^31,32^. These results indicated that as a template, 3D-DPFS can set a foundation for the preparation of 3D fibrous biomineralization scaffolds for 3D cell culture and osteogenic cell differentiation.

### Cellular proliferation, infiltration, and morphological analysis

**Figure 6:**
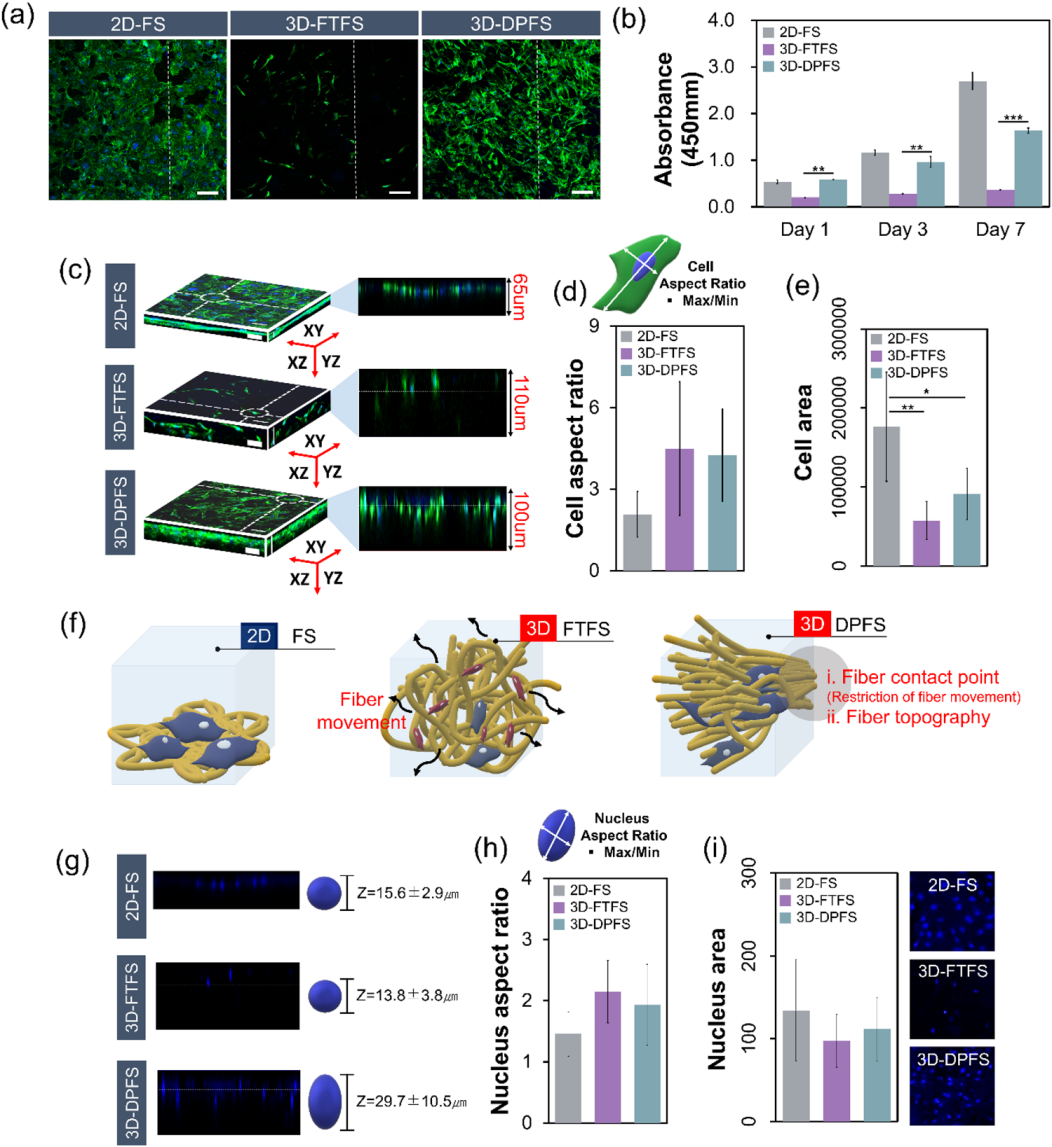
Cell morphologies on the different scaffolds and its quantitative analysis. (**a**) Confocal images showing proliferation of MC3T3-E1 cells after 7 days of culture (scale = 100 μm). (**b**) Viability assessment of MC3T3-E1 cells cultured for 1, 3, and 7 days in vitro (n = 3). (**c**) 3D orthogonal views (XY, XZ, and YZ) and its infiltration expressing the adhesion morphology of MC3T3-E1 cells. Quantitative analysis for (**d**) cell aspect ratio and (**e**) cell area. (**f)** Schematic diagrams showing cell adhesion morphology on 2D-FS, 3D-FTFS, and 3D-DPFS. (**g**) The orthogonal and cross-sectional z-stack nuclear morphology together with the mean nuclear thickness in cultured MC3T3-E1 cells on 2D-FS, 3D-FTFS, and 3D-DPFS. Quantitative analysis for (**h**) nucleus aspect ratio, (**i**) nucleus area.

We compared the adhesion, proliferation, and infiltration of pre-osteoblastic MC3T3-E1 cells on the 2D membrane and the two 3D fibrous scaffolds. To determine the effect of matrix guidance on cell proliferation, cells were cultured on the scaffolds and then evaluated with the cell counting kit-8 (CCK-8) (Fig. 6b). The 2D-FS group exhibited significantly high cell activity on day 7, while 3D-FTFS and 3D-DPFS were relatively lower than those of the 2D-FS group. This is because excessive movement of the fibers impedes cell attachment and fiber elongation due to the lack of contact points between the fibers of 3D-DPFS and 3D-FTFS fabricated based on fiber repulsion. However, the structure of the dianthus caryophyllus mimicking 3D-DPFS significantly accelerated cell proliferation and even offset the decrease in cell viability caused by initial attachment failure. At 7 days after culture, cells showed a more than four-fold increase in proliferation on 3D-DPFS compared to 3D-FTFS. Additionally, the high porosity and bottom-up hierarchical 3D porous structure in the 3D-DPFS largely improved cellular infiltration through more openly interconnected pores, which is an essential feature for efficient nutrient and waste exchange in and out of the fiber constructs, which led to the higher survival and proliferation of cells^33^. Loh et al., reported that 3D scaffolds are generally highly porous with an interconnected pore network to facilitate diffusion of oxygen and nutrient, and waste removal. They also revealed that the 3D scaffold serves to mimic the actual in vivo microenvironment in which cells interact according to mechanical and topographical cues obtained from the surrounding 3D environment^34^.

We visualized cell morphology and penetration into the scaffold via confocal laser microscopy (CLM) to confirm the ability of cells to penetrate the scaffold (Fig. 6a and c). On 2D-FS scaffolds, the cells appeared to spread out with a rather planar or 2D configuration by extending filopodia along random oriented fibers. Our experimental results showed that cells on 3D-FTFS scaffolds had a rounder shape and less cell spreading. This indicates poor cell adhesion and cell-scaffold interactions due to the hydrophobic property of PCL. In contrast, cells on 3D-DPFS scaffolds were elongated with a spindle-shaped morphology, indicating positive cell-scaffold interactions^35^. The reconstructed CLM images indicate that the cell proliferation and infiltration were restricted to a small range (approximately 65 μm) in the z direction of the 2D-FS surface (Fig. 6c). An important problem with 2D-FS is the poor infiltration of cells into their 3D structure^36,37^. Although the 2D-FS showed significant cell proliferation as compared to the 3D-DPFS and 3D-FTFS, the penetration of cells in the 2D-FS was not demonstrated (Fig. 6b and c). In contrast, cells seeded on 3D-DPFS and 3D-FTFS were shown to migrate much deeper, to a depth of about 100–110 μm (Fig. 6c). Additionally, cells cultured on 3D-DPFS and 3D-FTFS have different cell shapes and arrangements than cells on 2D-FS as shown in Fig. 6d and e. Cells in 3D-DPFS and 3D-FTFS showed increased cell aspect ratio and decreased cell area compared to 2D-FS (Fig. 6d and e). Cells in 3D-DPFS and 2D-FS presented a spindle-shaped morphology extending along the direction of the fiber. However, on 3D-FTFS, cells displayed short microfilaments that adhered to the fiber layer along a random network but failed to elongate (Fig. 6a and e). Cells showed similar behavior in the regulation of nuclear shape in response to changes in cell shape. The nuclei of the cells on 3D-DPFS demonstrated a thickness of 29.7 ± 10.5 μm, while 2D-FS and 3D-FTFS showed moderate thicknesses of 15.6 ± 2.9 μm and 13.8 ± 3.8 μm, respectively (Fig. 6g-i). Our results regarding cell aspect ratio and area indicate that the dimension and topography of scaffolds have an important impact in directing the degree of elongation and alignment, consistent with contact guidance phenomena^38^. Han et al., demonstrated that most existing electrospun nanofibers are in the form of tightly packed 2D membranes, which have the intrinsic disadvantages of limited cell penetration, limited nutrient diffusion, and unsatisfactory thickness. The research team fabricated three types of 3D ENF-S using different approaches, which are categorized as electrospun nanofiber 3D scaffolds, electrospun nanofiber/hydrogel composite 3D scaffolds, and electrospun nanofiber/porous matrix composite 3D scaffolds. As a result, new functions and properties of the fabricated 3D scaffolds, such as promoted cell infiltration, 3D fiber structure, improved mechanical properties, and tunable degradability, were identified to meet the requirements of tissue engineering scaffolds^39^. This 3D-DPFS mimicking dianthus caryophyllus exhibits increased cell proliferation and high penetration by reducing fiber migration and exhibiting high spatial interconnection, increase of fiber density, and controlled alignment (Fig. 6f). This approach presents great promise for the design of cell-permeable scaffolds for bone tissue engineering.

### Osteogenic differentiation evaluation of MC3T3-E1 cells

**Figure 7:**
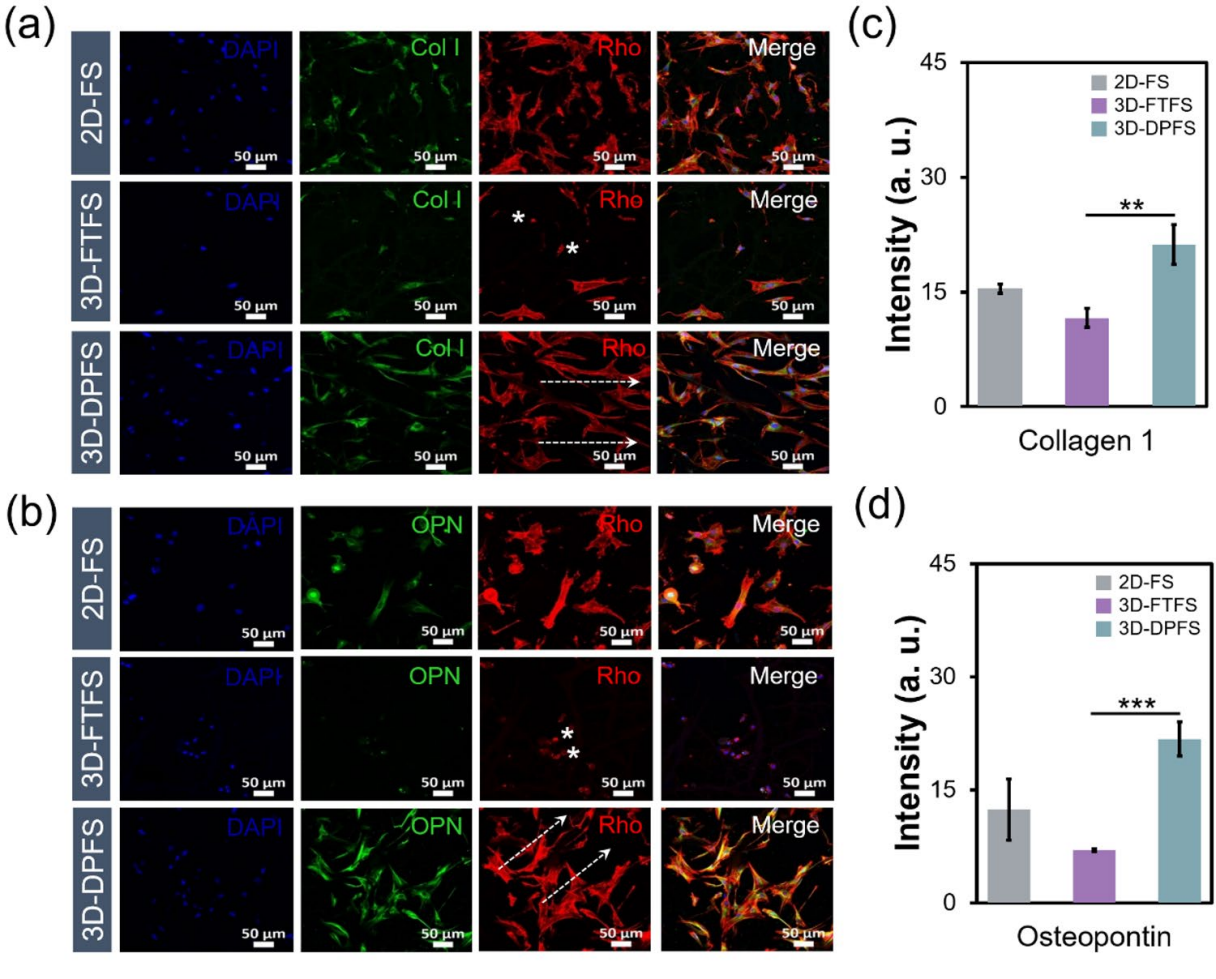
Osteogenic differentiation potential of 2D and the two 3D scaffolds. (**a**, **c**) Immunofluorescence images and (**b**, **d**) quantification graphs of MC3T3-E1 cells cultured for 3 days on 2D-FS, 3D-FTFS, and 3D-DPFS (n = 3).

To confirm the capability of as-spun 2D-FS, 3D-FTFS, and 3D-DPFS in guiding the differentiation of MC3T3-E1 osteoblasts in vitro, osteogenic markers were observed via immunostaining at day 3 (Figs. 7a–d). The expression of collagen type 1 (Col I) and the osteoblast-specific extracellular matrix proteins, osteopontin (OPN), are reliable indicators of osteogenic differentiation^40,41^. Also, OPN is the most important bone biomarker responsible for the initiation and maintenance of bone mineralization^42,43^. We found that cells cultured on 3D-DPFS expressed significantly higher Col I and OPN compared to those on other scaffolds. Differentiation of pre-osteoblastic cells cultured on the surface of biomaterials may be affected by various factors such as the chemical composition of the scaffolds, fiber density, surface roughness, and even the stiffness (Young’s modulus) of the materials^44,45^. Therefore, the transient and specific response of 2D-FS, 3D-FTFS, and 3D-DPFS with the same chemical composition to specific markers of osteogenesis may be attributed to the microstructural and mechanical properties of the substrates. Importantly, the 3D-DPFS provides an instructive microenvironment for 3D cell culture through cell-to-scaffold interaction. In summary, an innovative approach for developing a dianthus caryophyllus-mimetic nano-and micro-patterned fibrous structure was applied to prove the potential application of the designed scaffold for bone regeneration. The fabricated 3D-DPFS was observed using SEM, which revealed a porous structure with aligned cotton-type fibers. Due to this prominent feature, the 3D-DPFS not only possesses excellent mechanical properties but also superior bioactivity due to the induction of precipitation of bone-like apatite minerals. Compared with the 2D membrane and previous fluffy type mesh, the 3D-DPFS prolonged the reconstruction period and enhanced cell infiltration and proliferation. Further, the evaluation of immunofluorescence staining via osteogenic proteins also showed the potential of the 3D-DPFS as an ideal material for bone tissue engineering applications.

## Conclusions

In tissue engineering, the use of electrospinning is a valuable choice to design and fabricate biomimetic micro/nanostructures for cell culture and new tissue formation in three dimensions. Here, we report the fabrication of scaffolds consisting of a highly porous structure with flower-like morphology by utilizing a sharply inclined array collector included point electrode (SIAC-PE). The point electrode effect led to the hierarchical deposit of aligned fibers on the surface of the collector. Moreover, the formation of this 3D structure was found to depend on the viscosity of the solution which varies with the stirring time of LA inside the PCL solution. The fiber density, fiber diameter, and fiber orientation of the formed 3D structure were confirmed by scanning electron microscopy. In addition, the compressive mechanical property of the 3D scaffold was studied. It possessed great elastic properties. The biocompatibility of the 3D scaffold was evaluated using pre-osteoblastic MC3T3-E1 cells. The 3D structure with deeply interconnected pores and aligned fibers exhibited enhanced cell infiltration, proliferation, and differentiation. The results demonstrated that such a production method of the 3D fibrous PCL scaffold has great potential for biomaterial matrices preparation for tissue engineering.

### Statistical analysis

Unless specified otherwise, all data are represented as mean ± standard deviation and analyzed using SPSS version 16.0 software. Differences between the two groups were analyzed by a Student’s *t*-test. One-way analysis of variance followed by a post-hoc Tukey test was used for multiple comparisons among more than two groups. *p* < 0.05 indicated statistical significance.

### Supplementary Information

Supplementary Figures.

## Data Availability

Research data are shared if requested to corresponding author.
