# Supplementary material for: 3D cotton-type anisotropic biomimetic scaffold with low fiber motion electrospun via a sharply inclined array collector for induced osteogenesis
Source: Sci Rep. 2024 Mar 28;14:7365. doi: 10.1038/s41598-024-58135-2 (PMC10978854; doi:10.1038/s41598-024-58135-2)
Supplement: Supplementary file 1 — Supplementary Figures. [file 41598_2024_58135_MOESM1_ESM.docx]

**Supplementary Information (SI)**

**3D cotton-type anisotropic biomimetic scaffold with low fiber motion electrospun via a sharply inclined array collector for induced osteogenesis**

Sun Hee Cho^1^, Soonchul Lee^2*^, Jeong In Kim^2*^,

*^1^Department of Bionanotechnology and Bioconvergence Engineering, Graduate School, Jeonbuk National University, Jeonju 54896, Republic of Korea*

*^2^ Department of Orthopaedic Surgery, CHA Bundang Medical Center, CHA University School of Medicine, 335 Pangyo-ro, Bundang-gu, Gyeonggi-do, Republic of Korea*

*Corresponding authors

E-mail: lsceline78@gmail.com (S.C.Lee), codl3311@naver.com (J.I.Kim)

**Supplementary Figures**


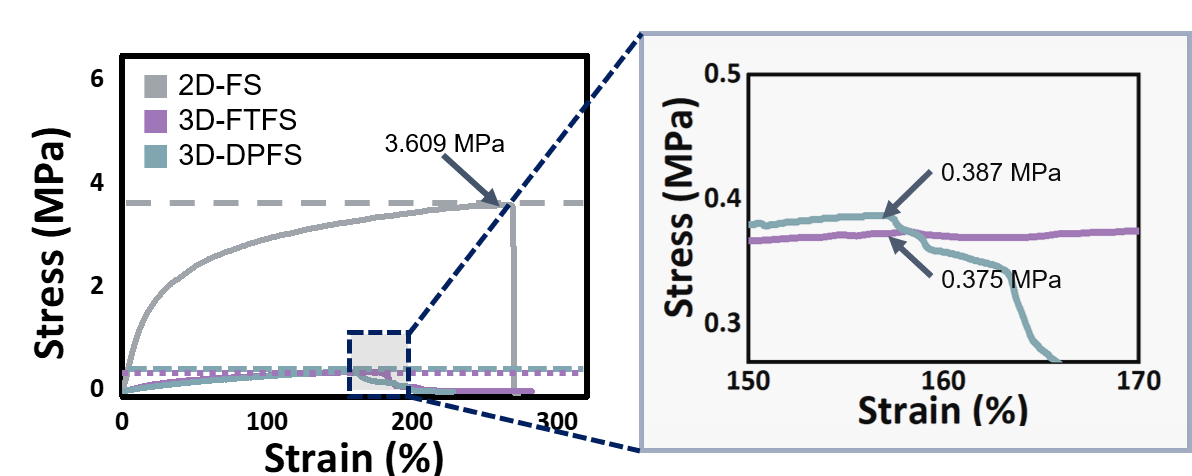


**Figure S1.** Tensile stress-strain curves of 2D-FS, 3D-FTFS, and 3D-DPFS.


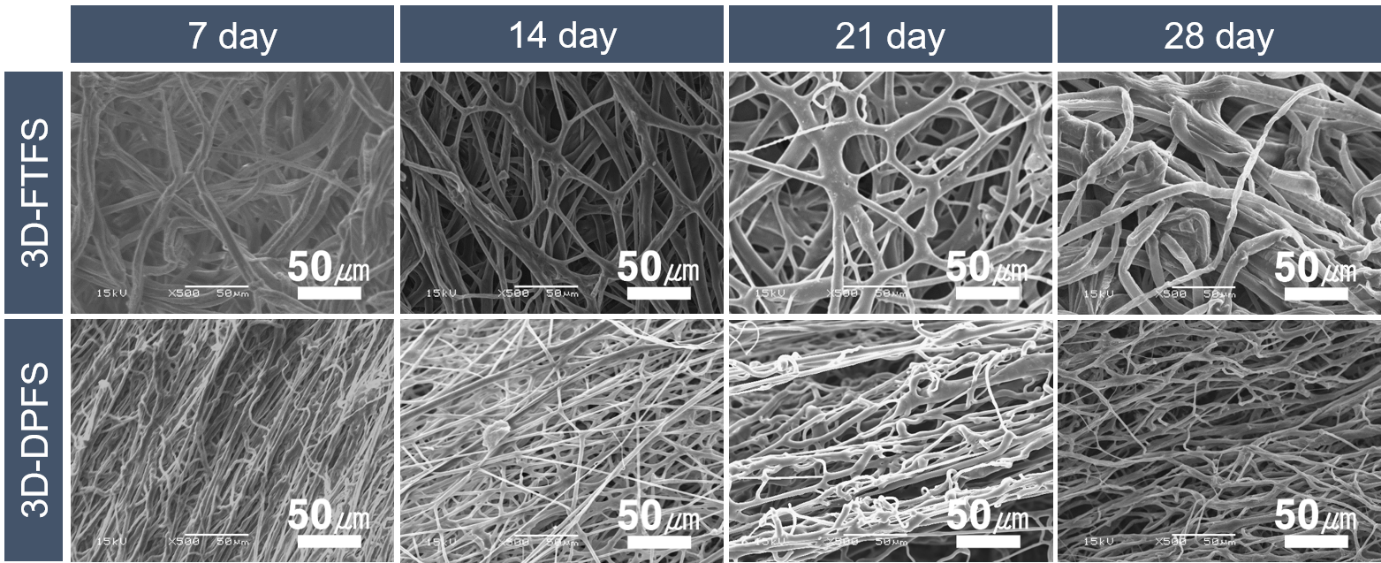


**Figure S2.** SEM images showing degradation behaviors of 3D-FTFS and 3D-DPFS.
